# Qualitative process evaluation of a new short-hospital-stay model with a general medicine focus in Germany (STATAMED): Study protocol

**DOI:** 10.1371/journal.pone.0327556

**Published:** 2025-07-10

**Authors:** Katharina van Baal, Juliane Poeck, Clarissa Maria Lemmen, Wiebke Böhne, Johannes Jahn, Eva-Maria Wild, Nils Schneider

**Affiliations:** 1 Institute for General Practice and Palliative Care, Hannover Medical School, Hannover, Germany; 2 AOK Rhineland/Hamburg – The Health Insurance Fund, Health Management Division, Health Care Innovations Department, Düsseldorf, Germany; 3 Health Insurance Fund AOK Lower Saxony – Division Policy, Research and Press, Hannover, Germany; 4 Hamburg Center for Health Economics, University of Hamburg, Hamburg, Germany; PLOS: Public Library of Science, UNITED KINGDOM OF GREAT BRITAIN AND NORTHERN IRELAND

## Abstract

**Background:**

The increasing number of elderly and multimorbid patients in need of care, coupled with a shortage of skilled health care professionals, poses significant challenges, especially in structurally underserved regions. STATAMED (funded by the G-BA Innovation Fund, 01NVF22103) represents a novel cross-sector care model emphasising short hospital stays with a general medicine focus for patients with (sub-)acute treatment needs. The intervention provides targeted, short-term inpatient care within STATAMED units, followed by rapid discharge and subsequent follow-up care in familiar environments. Key objectives include reduced emergency department visits, hospital stays and re-admissions, as well as the provision of needs-oriented health care. The broader evaluation comprises multiple quantitative and qualitative sub-studies. The present sub-study involves a qualitative process evaluation investigating the factors influencing acceptance, the degree of implementation and the impact on daily workflows and care structures at STATAMED sites.

**Methods:**

The sub-study will employ a combination of qualitative health services research methods (i.e., focus group discussions, individual interviews, participatory observations, discussion and information forums) to capture the perspectives and experiences of three main stakeholder groups: (1) patients and their relatives, (2) health care providers and (3) the general population. Data collected through protocols and transcripts will undergo qualitative content analysis to identify recurring themes and insights.

**Discussion:**

This sub-study seeks to elucidate the expectations, attitudes and needs of stakeholders, while assessing overall acceptance of the STATAMED model. Additionally, it aims to identify both facilitators and barriers to implementation, as well as the implications for work processes, collaboration and communication within daily care. The findings will inform strategies to optimise implementation, ensuring a successful transition to routine care. Given the political and public sensitivity surrounding health care system redesigns, particularly in rural areas, diverse stakeholder perspectives are incorporated.

**Trial registration:**

The study was registered with the German Clinical Trials Register (Deutsches Register Klinischer Studien; DRKS00033801; date of registration: 22 March 2024).

## Introduction

### Challenges in health care systems

Globally, and particularly in high-income countries, health care systems are facing significant challenges associated with ageing populations and the increasing prevalence of chronic diseases [1,2]. These trends are reshaping health care demand, with rural areas disproportionally affected due to demographic change and a concurrent withdrawal of health care providers [3–5]. In Germany, the population aged 65 and older currently stands at 18.3 million [6] and is projected to rise to 23.1 million by 2040, thereby further intensifying the demand for health care services [7]. Simultaneously, the number of individuals requiring long-term care continues to grow [8]. Elderly and multimorbid patients, often hindered by illness, immobility, limited social networks and a lack of access to adequately qualified non-physician health care professionals, frequently encounter barriers to appropriate outpatient care, as delivered by nurses, physiotherapists or nutritionists. This inadequacy drives many patients to hospitals, even when resource-intensive inpatient diagnostic and therapeutic treatment is unnecessary. Such admissions often lead to overdiagnosis within highly specialised hospital departments, resulting in extended stays yielding limited or no patient benefits [9]. The need for health care system reform to address the needs of elderly patients with chronic diseases is not a new concern [10]. However, the urgency for targeted solutions has grown, particularly in rural settings where challenges are compounded.

### Structural and political implications in Germany

The hospital sector in Germany is undergoing significant transformation, driven by the imperative to adapt to the needs of elderly and multimorbid patients, particularly in rural areas. Existing hospital structures are often ill-suited to this population, necessitating systemic reforms. The 2021–2025 coalition agreement emphasises the establishment of hospital planning standards based on service groups and care levels, while considering accessibility and demographic trends [11]. Key objectives of the proposed hospital reform include safeguarding and improved quality of care, comprehensive medical services and reduced administrative burdens. The current case-by-case payment model has imposed severe financial strain on hospitals in Germany, threatening the closure of many facilities. To ensure financial sustainability, the reform proposes a transition to fixed-rate payments for essential clinics, while shifting the focus from quantity to quality in patient care [12].

The challenges facing German hospitals are well-documented. In particular, a 2018 report from the German Council of Experts identified overcrowded emergency rooms, overworked staff and prolonged waiting times as critical issues [13]. These pressures have fuelled a trend towards larger clinics, thereby adversely affecting smaller care units, particularly in rural areas. To address these issues, transformation of the hospital landscape in Germany is crucial. Especially for elderly, multimorbid patients, regional short-stay units present a viable alternative to traditional inpatient hospital care, helping to alleviate the burden on emergency rooms while maintaining efficiency and accessibility. STATAMED may make an important contribution to this structural transformation, particularly through the active involvement of local stakeholders.

In the context of persistent skill shortages, accessible, low-threshold health care becomes increasingly important for optimising the use of medical and nursing resources. Costly and time-consuming processes caused by rigid sectoral boundaries must be avoided. STATAMED may address these challenges by reducing the reliance on traditional hospital beds and emphasising low-threshold care solutions, thereby alleviating pressure on other inpatient capacities and contributing to a more efficient health care system.

### Short-stay units as a potential answer to existing challenges

Short-stay units are being increasingly discussed as a potential solution to address the needs of elderly patients with chronic diseases [14]. Defined by specific admission criteria and a targeted length of stay, these units aim to improve care quality and cost-effectiveness [15] by offering tailored care with fewer resources. They are particularly well-suited for patients who do not require surgical intervention or highly technical diagnostics, making them a promising option for the delivery of high-quality of care to elderly patients with chronic diseases. Short-stay units can also provide accessible, resource-efficient health care in rural areas, and by preventing unnecessary inpatient stays, they may even reduce the risk of hospital-acquired conditions such as immobility [15]. However, while some studies have observed benefits such as increased patient satisfaction [14] and reduced lengths of stay with no adverse effects on mortality or re-admission [16], evidence on the overall impact of short-stay units remains inconclusive, due to methodological limitations [15].

### STATAMED study context

The restructuring of Germany’s hospital landscape has placed many existing facilities –particularly those in rural areas – at risk of closure. STATAMED offers a strategic response to this threat by repurposing at-risk hospitals into short-stay units featuring a general medical ward with approximately 15–25 beds. Through this transformation, small hospitals in rural areas, which are no longer financially viable due to low utilisation rates, may gain a renewed purpose. This reorganisation involves a phased reduction in the number of hospital beds and specialist departments and the planned closure of emergency departments.

To ensure appropriate care and avoid unnecessary hospital admission, the need for short-stay inpatient treatment is determined through a cross-sectoral assessment, including a structured admission interview conducted by a STATAMED physician in collaboration with the referring entity (e.g., physician, emergency paramedic, residential care facility nurse, outpatient nursing service). STATAMED bridges outpatient and inpatient care through several mechanisms: (1) communication and collaboration across sectors and professional groups, (2) interdisciplinary and cross-sectoral treatment planning, (3) case management (involving a case manager) to coordinate cross-sectoral and cross-organisational care and (4) mobile nursing (through a ‘Flying Nurse’), where necessary and indicated by the STATAMED physician, to provide up to four weeks of follow-up care in patients’ homes following discharge. Furthermore, STATAMED offers cross-sectoral networking among medical professionals and specialists. These experts provide their services either through telemedicine or in direct collaboration with STATAMED.

## Aims

The primary objective of STATAMED is to establish and evaluate a new cross-sectoral, short-stay inpatient general medical care model. This innovative intervention targets patients with (sub-)acute conditions who require medical treatment but do not need resource-intensive inpatient diagnostics or therapies. The model aims to reduce reliance on emergency care, shorten hospital stays, prevent readmission and deliver needs-oriented health care.

STATAMED is being scientifically evaluated through multiple sub-studies conducted by consortium partners. The present study protocol refers to the qualitative process evaluation, which will investigate the implementation of the care model and the factors that facilitate or hinder its acceptance and degree of implementation. Additionally, the sub-study will assess the impact of the new model on daily work routines and care structures at STATAMED sites. The findings will be integrated with the results of the summative and formative evaluations over the course of the project to generate evidence to support the transition of STATAMED into standard care.

## Research questions

The present study protocol refers to the qualitative process evaluation sub-study, addressing three central research questions:

1. Which factors influence the acceptance of the new care model among various stakeholders (i.e., health care providers, patients and their relatives, the general population) and how do these factors affect the degree of implementation?
2. How does the new care model change daily work processes at STATAMED sites, as perceived by health care providers and referring entities?
3. To what extent does the implementation of STATAMED affect care structures at STATAMED sites, from the perspective of health care providers and referring entities?

## Methods and design

STATAMED is a 45-month project (01.07.2023–31.03.2027) funded by the Innovation Fund of the Federal Joint Committee (Grant No.: 01NVF22103). The funding body has no influence on the design of the study, the collection, analysis or interpretation of the data or the manuscript preparation. The reporting follows the SPIRIT Statement (Standard Protocol Items: Recommendations for Interventional Trials; S1 Table).

### Setting

STATAMED is a pilot project conducted across six regions in both rural and urban areas of Germany (Lower Saxony, Hamburg, North Rhine-Westphalia). The qualitative process evaluation will focus on three rural STATAMED sites (Bad Gandersheim, Norden, Sulingen) and one urban site (Essen).

### Study design

STATAMED is an interventional study aimed at implementing a needs-oriented transformation of patient pathways via a cross-sector, short-hospital-stay care model with a general medicine focus for patients with (sub-)acute treatment needs. The study employs a multi-centre, multi-method evaluation approach. The present protocol specifically describes the qualitative process evaluation, which will adopt a multi-perspective approach encompassing prospective, retrospective and observational components. Various qualitative methods will be used to determine model acceptance among different target groups, identify factors influencing the implementation of the care model and examine changes in the daily health care workflows and care structures at STATAMED sites.

### Study phases

The overall STATAMED project is divided into three phases over its 45-month duration: a preparatory phase (July 2023–March 2024), an interventional phase (April 2024–March 2026) and an evaluation phase (April 2026–March 2027).

**Fig 1:**
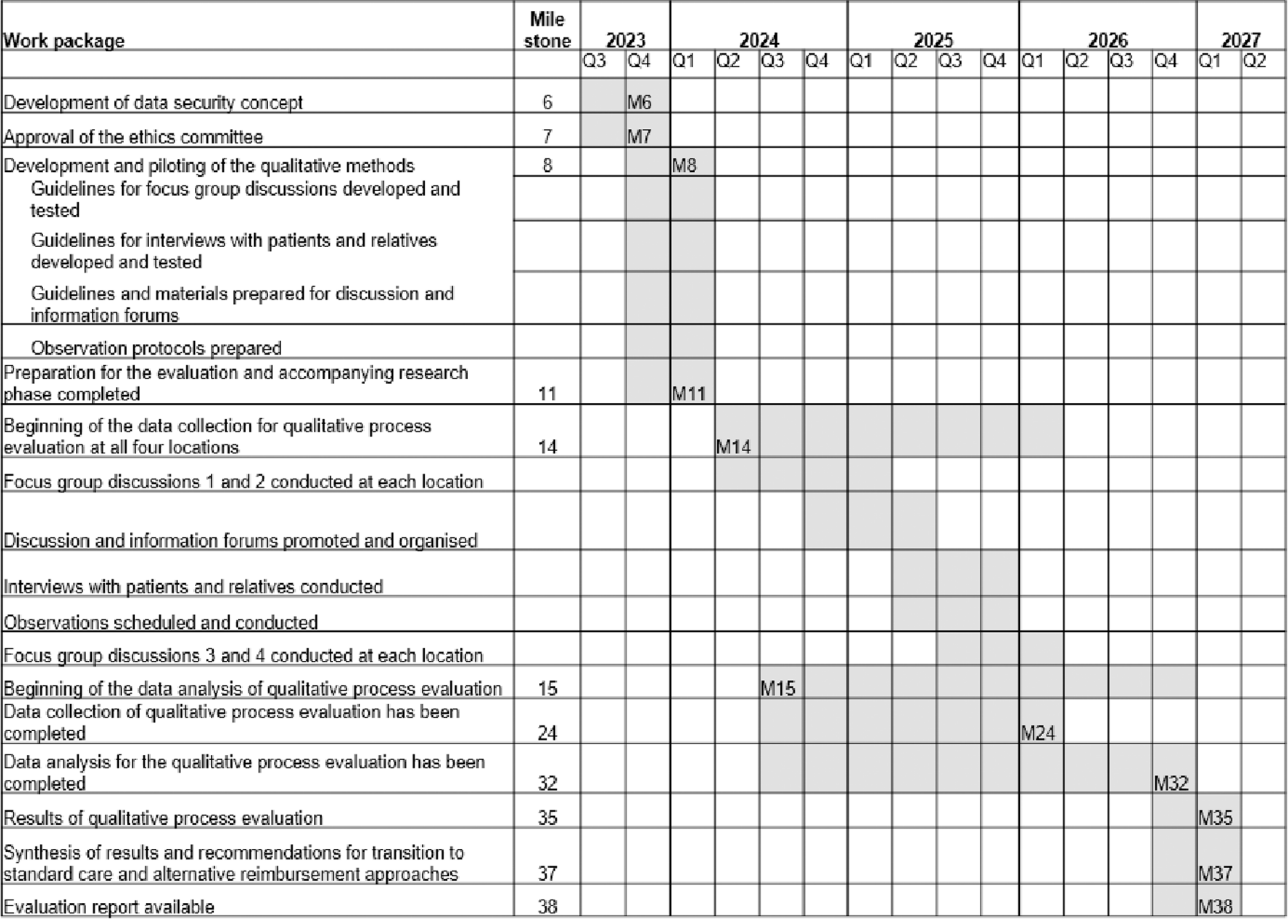
WPs and milestones of the qualitative process evaluation.

The qualitative process evaluation sub-study extends over the entire project period, capturing data from all phases. **Fig 1** outlines the key work packages (WPs) and milestones. Data collection in currently ongoing and will presumably end in March 2026. The results of the qualitative process evaluation will be available in March 2027.

#### Preparatory phase (1).

During the preparatory phase (July 2023–March 2024), foundational tasks for the qualitative process evaluation were completed. Key milestones included the development of a data security concept (M6), confirmation of ethical approval (M7) and preparation for data collection (i.e., creation of recruitment materials, development and piloting of the qualitative instruments) (M8). The phase concluded with final preparations for the evaluation and the accompanying research (M11).

#### Interventional phase (2).

The interventional phase (April 2024–March 2026) marks the beginning of data collection for the qualitative process evaluation during the model implementation at the STATAMED sites (M14). Key activities include: focus group discussions with health care providers, individual interviews with patients and their relatives, participant observation within STATAMED units, and discussion and information forums. By the end of this phase, data collection will be completed (M24). Data analysis will begin shortly after the first data assessments are conducted (M15).

#### Evaluation phase (3).

The evaluation phase (April 2026–March 2027) will involve the completion of the analyses and synthesis of the findings. Key milestones will include finalising the data analysis (M32), summarising the results of the qualitative process evaluation (M35), integrating these results with findings from other project partners to develop recommendations for transitioning STATAMED into routine care (M37) and preparing and disseminating a final report to project partners and the public (M38).

The subsequent sections provide detailed descriptions of the study population, recruitment strategies, sample sizes and anticipated outcomes specific to the qualitative process evaluation. Details of other sub-studies are documented separately and can be accessed via the German Clinical Trials Register (Deutsches Register Klinischer Studien, DRKS00033096).

### Study population and recruitment

The present sub-study aims to capture the perspectives of health care providers, patients and their relatives, and the general population at the STATAMED sites. Participation is open to individuals of all genders and ethnic backgrounds who are at least 18 years old. A purposive sampling strategy will be employed to ensure a diverse range of perspectives relevant to the research questions [17]. The sample size will be guided by the principle of theoretical saturation, whereby data collection will continue until no new insights on the research question are expected [18]. The recruitment phase is currently ongoing and is expected to last from September 2024 until March 2026.

Further details on the inclusion and exclusion criteria, targeted sample sizes, data collection timelines and recruitment processes for the four WPs in the qualitative process evaluation are described below.

#### WP 1: Focus group discussions with health care providers.

Target group 1 encompasses health care providers actively involved in STATAMED, including medical and nursing staff working in STATAMED units, case managers, mobile ‘Flying Nurses’ and referring entities at the four STATAMED sites.

Target group 2 comprises health care providers who are not (yet) involved in STATAMED, including clinicians from other hospitals, general practitioners and nursing staff from outpatient care services and inpatient care facilities located in the four target regions.

Focus group discussions will be conducted at two time points (t0: 10/2024–03/2025; t1: 01/2026–04/2026). At each time point, two focus groups per target region are planned with both target groups, with each group comprising 8–10 participants. Consequently, a total of 64–80 participants per time point are anticipated (16–20 participants per target region). Each focus group will last approximately 120 minutes. Recruitment will be conducted via direct invitation (by email, fax or telephone), with all written invitations distributed by case managers within the STATAMED units. Contact details of the health care providers in regional health care networks will be shared via a trust centre and supplemented by manual research.

#### WP 2: Individual interviews with patients and relatives.

The target group for the individual interviews consists of patients treated in a STATAMED unit and their relatives, provided the latter are identified by patients as their primary caregiver or person of trust. Participation requirements include an interest in the study, adequate health to participate and sufficient German language proficiency to partake in an interview. Between September and December 2025, individual interviews will be conducted with 8–10 patients and their relatives in each target region. Recruitment will occur through written invitation letters distributed by case managers in the STATAMED units.

#### WP 3: Participatory observation.

The third component of the qualitative process evaluation involves the participatory observation of daily processes and procedures at the four STATAMED units. On-site observations, lasting approximately 3–4 hours per unit, are planned for October 2025. Invitations to participate will be sent in writing, and appointments at the STATAMED units will be scheduled via telephone or email. Pilot/case managers at the STATAMED units will facilitate access and distribute the invitation letters.

#### WP 4: discussion and information forums.

The discussion and information forums aim to engage the target groups from WPs 1–3, as well as members of the general population in the four target regions. One discussion and information forum will be held at each location between April and July 2025, with approximately 50–60 participants per forum. Each event will last 2–2.5 hours. Multiple strategies will be employed to reach this broad audience: (1) invitations will be distributed via letter (email, fax, post), information flyers and social media platforms; (2) invitations will be shared through STATAMED institutions (via contact persons), health care providers and networks (via large distribution lists), community foundations and consortium partner contacts; and (3) flyers and posters will be displayed in STATAMED units, medical supply stores, pharmacies, physiotherapy practices, citizen offices and city libraries.

### Outcomes

The qualitative process evaluation is designed to generate evidence on the following secondary outcome measures:

- awareness and acceptance of the STATAMED care model among various target groups;
- degree of STATAMED implementation, including factors that promote or hinder successful implementation; and
- changes in daily workflows within STATAMED institutions.

The primary outcomes (e.g., re-hospitalisation, length of inpatient stay) are being evaluated by another research partner (DRKS00033096) and are not included in this sub-study.

### Ethics and data protection

The study received ethical approval from the Ethics Committee of the Hannover Medical School on 6 December 2023 (No.: 11149_BO_S_2023; see S2 Protocol and S3 Protocol) in accordance with the Declaration of Helsinki. All study participants will be provided with detailed verbal and written information about the project objectives and data protection protocols before confirming their participation. Participation will be voluntary and require signature of a written consent form. Participants may refuse or withdraw their consent to participate at any time and without providing a reason.

Each participant will be assigned a unique code to ensure data pseudonymisation. The code list will be stored securely and separately from the data collection documents, and access to the study materials will be restricted to members of the research team. Any significant amendment to the study protocol or interventions will be submitted to the Ethics Committee of the Hannover Medical School for prior approval.

### Data analysis

Qualitative data (WPs 1, 2 and 4): Data collected from focus group discussions, individual interviews, and discussion and information forums will be audio-recorded, transcribed and pseudonymised. The resulting transcripts and protocols will be analysed using qualitative content analysis, as described by Mayring [19] or Kuckartz [20], using the software MAXQDA. Socio-demographic data (e.g., age, gender, occupation) will be collected via a short questionnaire and analysed descriptively using SPSS.

Qualitative and quantitative data (WP 3): Data from participatory observations will be recorded in structured observation protocols and analysed according to data type. Qualitative data (e.g., open comments, conversational notes, recorded/transcribed discussions) will undergo content analysis according to Mayring [19] or Kuckartz [20], Quantitative data (e.g., frequency counts, Likert scale ratings) will be analysed descriptively. Findings from both data sources will be synthesised and summarised.

## Discussion

### Expected results

The STATAMED project is anticipated to yield valuable insights into the effects of implementing an innovative, cross-sector, short-hospital-stay care model with a general medicine focus. For patients, potential benefits include reduced emergency care, fewer hospitalisations and prevention of medical overuse or inappropriate care. For inpatient facilities, improvements may include relief for emergency departments and more efficient distribution of patients based on complexity and care needs. For health care practitioners, transformation of the hospital landscape could optimise the allocation of medical and nursing resources, addressing the challenges of a skilled worker shortage. Finally, general practices and nursing facilities might benefit from improved cross-sectoral communication and stronger collaboration within the target regions.

The sub-study will generate different perspectives on the acceptance of the STATAMED care model among health care providers, patients and their relatives, and the general population across the four target regions. The multi-methods approach will ensure that local sensitivities and public sentiments are addressed, and the participatory involvement of stakeholders will strengthen the transformation process.

Findings on the acceptance, needs, perspectives and challenges of implementing STATAMED will serve to optimise the implementation process, facilitate transferability to other regions and enable a successful transition of the STATAMED care model to routine care.

Understanding the factors critical to the successful implementation of STATAMED is a need that extends beyond the scope of the project, itself. As mentioned above, the challenges that STATAMED aims to address are not unique to Germany, but relevant to many countries. Thus, this evaluation of the implementation process will generate valuable insights into the success factors and potential pitfalls that could inform the design and execution of similar projects internationally.

### Study risks

Access to health care providers in inpatient care may pose challenges due to varying attitudes towards and experiences with scientific research, as well as constraints on participants’ time and capacity. However, recruitment of patients and their relatives is not expected to encounter significant barriers, as these groups will be mainly recruited by case managers during personal interactions. To mitigate recruitment challenges among health care providers, the project will systematically involve local Associations of Statutory Health Insurance Physicians. Recruitment efforts will also include: (1) the provision of concise and accessible information on participation, (2) support from consortium partners in the dissemination of recruitment materials and (3) regional engagement with professional networks in each STATAMED target region.

No disadvantages, harm or violations of personal rights are anticipated for study participants. The time commitment for participants will vary according to the research method, with the following durations anticipated: (1) 120 minutes for focus groups, (2) 30–60 minutes for individual interviews, (3) 180–240 minutes for participatory observations of daily work (not requiring direct participant engagement) and (4) 120–150 minutes for discussion and information forums.

Experience has shown that technically and economically sound reforms may face resistance if local sensitivities and stakeholder concerns are not taken into account. Therefore, particular attention will be given to these ‘soft’ factors, ensuring all stakeholders are actively engaged in the transformation process through a participatory approach. It is recognised that urban and rural areas may present distinct challenges affecting the acceptance and implementation of the STATAMED model. For example, urban areas may face unique obstacles due to their higher availability of health care services compared to rural regions. To ensure the transferability of the findings to such areas, the qualitative process evaluation will include an urban target region (Essen) while retaining a primary focus on rural sites.

### Innovative potential and benefit

The qualitative process evaluation will employ a diverse range of methods (focus group discussions, individual interviews, participant observation, discussion and information forums) to gather insights from different target groups and engage them in the transformation process. In addition to recording both inhibiting and promoting factors during the intervention, the qualitative process evaluation will aim to raise public awareness and generate actionable insights to support the transition of the STATAMED care model within the general population. By explicitly involving key stakeholders – actual users (patients), health care providers and potential users (the general population) – the qualitative process evaluation will ensure that diverse perspectives shape the transformation process.

### Dissemination and implementation

Implementation of the new cross-sector, short-hospital-stay care model with a general medicine focus necessitates change at multiple levels while offering significant insights. The evaluation carried out in this sub-study will provide key findings on model acceptance among the general population and other stakeholders within the target regions, particularly with regard to the re-organisation of hospital structures and the potential integration of the STATAMED model into routine care. Detailed insights into the indication profiles, qualification requirements for health care providers and the minimum infrastructure needed for STATAMED units will facilitate the model’s transferability to a wide range of regions and health care settings. In the long term, the STATAMED model could provide a viable alternative to the closure of small district hospitals, especially in structurally disadvantaged regions. This re-organisation of medical services into a more integrated and needs-oriented care model has the potential to enhance acceptance among the general population while addressing critical health care challenges.

## Supporting information

S1 Table SPIRIT checklist. (PDF)

S2 Protocol Study protocol ethics vote version 1. (PDF)

S3 Protocol Study protocol ethics vote version 2. (PDF)
