# Supplementary material for: Qualitative process evaluation of a new short-hospital-stay model with a general medicine focus in Germany (STATAMED): Study protocol
Source: PLoS One. 2025 Jul 10;20(7):e0327556. doi: 10.1371/journal.pone.0327556 (PMC12244472; doi:10.1371/journal.pone.0327556)
Supplement: S2 Protocol — (PDF) [file pone.0327556.s002.pdf]

Study protocol

Transformation of the patient pathway through a cross-sector short inpatient general medicine-orientated care model (STATAMED) - Qualitative process evaluation

1. Responsibilities

1.1 Project management

Study management:

Prof Dr med Nils Schneider, MPH

Institute for General Practice and Palliative Medicine, OE 5440 Carl-Neuberg-Straße 1, 30625 Hannover

Phone: +49 511 532-6530

E-Mail: Schneider.Nils@mh-hannover.de

Deputy Director of Studies:

Dr rer. biol. hum. Katharina van Baal

Institute for General Practice and Palliative Medicine, OE 5440 Carl-Neuberg-Straße 1, 30625 Hannover

Phone +49 511 532-4506

e-mail: vanBaal.Katharina@mh-hannover.de

1.2 Project realisation

Research assistant:

Juliane Poeck, M.Sc.

Institute for General Practice and Palliative Medicine, OE 5440 Carl-Neuberg-Straße 1, 30625 Hannover

Phone: +49 511 532-4380

e-mail: Poeck.Juliane@mh-hannover.de

We expect to hire another research assistant in the 2nd quarter of 2024.

1.3 Project funding

This project is funded by the G-BA Innovation Fund (funding code: 01NVF22103) for a period of 45 months.

2. Objectives and justification of the study

2.1 Background

There are currently around 18.3 million people over the age of 65 living in Germany (Federal Statistical Office, 2023a). This number is estimated to increase to 23.1 million by 2040 and will require more and more medical care (Nowossadeck, 2012). This is particularly true for rural regions. At the same time, the number of people in need of care in Germany is rising (Federal Statistical Office, 2023b). The over-80s in particular are contributing to the high increase in patients in emergency departments (Groening et al., 2017). Older, multimorbid people in the outpatient sector are often unable to receive the care they need due to their illnesses, immobility, lack of familiarity with modern means of communication, insufficient social network or lack of access to sufficiently qualified non-medical professional groups. As a rule, patients are admitted to hospital even though resource-intensive inpatient diagnostic and therapeutic treatment is not necessary. Inpatient admission can lead to these patients being "reflexively" overdiagnosed in increasingly specialised departments of larger hospitals, often with long lengths of stay and without benefit for the patient (Bertelsmann Stiftung, 2019).

STATAMED aims to counteract this as a new care model with regional short-stay general medical care. A cross-sector assessment of the need for inpatient treatment by means of a structured referral discussion and an on-site assessment by the 'flying nurse' with special competences is intended to avoid premature hospital admissions. The STATAMED concept is given additional importance by the necessary structural change in the inpatient sector, especially in rural areas. The 2021-2025 coalition agreement envisages future standards for hospital planning based on service groups and care levels, taking into account accessibility and demographic trends in the population. Similarly, the German Council of Experts for the Assessment of Developments in the Healthcare System stated back in 2018 that overcrowded emergency departments with hours-long waits and overworked staff in hospitals are a major problem in the healthcare system, meaning that hospital landscapes need to be restructured. In this respect, STATAMED can make an important contribution to this structural process, especially as the local stakeholders are fully involved.

2.2 Study objective and questions

The overarching aim of STATAMED is to establish a new cross-sector, short-stay, general medicine-orientated form of care in the healthcare system. Patients with (sub-)acute complaints who require medical treatment without having to be transported to the emergency room should benefit from this. The reorganisation of hospitals will initially

is being trialled at 6 locations in Lower Saxony, Hamburg and the Rhineland. The aim of this new care service is to protect patients from overuse and inappropriate care, reduce the number and duration of hospitalisations and the use of emergency care, among other things.

STATAMED is being evaluated in several sub-studies at population and organisational level with the participation of various consortium partners. The HCHE is leading the evaluation and is primarily conducting the questionnaire- and routine data-based outcome evaluation, while the HCB is conducting the health economic evaluation.

The following exploratory research questions are to be answered as part of the qualitative process evaluation to be carried out by MHH:

I. What factors influence the acceptance of different stakeholders (care providers, patients, residents/relatives, population) and the degree of implementation of the intervention?
II. To what extent is everyday working life at STATAMED facilities changing?
III. To what extent does the implementation of STATAMED facilities influence the care structures in the respective target regions?

2.3 Own preparatory work on the study topic

The project consortium is multi-professional and interdisciplinary and consists of stakeholders from self-administration, healthcare and science. Under the project management of AOK Rheinland/Hamburg, the consortium partners are AOK Niedersachsen, the Institute for Health Care Business GmbH (HCB), the Hamburg Centre for Health Economics at the University of Hamburg (HCHE), the University Medical Centre Hamburg-Eppendorf, the Catholic parish of St. Maximilian Kolbe, the Klinikverbund Landkreis Diepholz gGmbH, the Klinikverbund Landkreis Diepholz gGmbH, the Klinikverbund Landkreis Diepholz gGmbH, the Klinikverbund Landkreis Diepholz gGmbH and the Klinikverbund Landkreis Diepholz gGmbH. Maximilian Kolbe, Klinikverbund Landkreis Diepholz gGmbH, SKH Stadtteilklinik Hamburg GmbH, St. Augustinus Gelsenkirchen GmbH, Ubbo-Emmius-Klinik Norden, Universitätsmedizin der Georg-August-Universität Göttingen, VivaQ Medizinisches Versorgungszentrum Mümmelmannsberg GmbH and the Institut für Allgemeinmedizin und Palliativmedizin der Medizinischen Hochschule Hannover (MHH). Overall, the consortium has extensive experience in health system and health care research, including all relevant expertise in qualitative and quantitative research methods.

The Institute of General Practice and Palliative Medicine has many years of healthcare experience at the interface between university outpatient clinics and GP care and is scientifically recognised in healthcare research (quantitative and qualitative methods). It also has expertise in projects on GP care, patient and family centred care, health system research and new forms of care.

2.4 Expected benefit

The results will provide current findings on how the new STATAMED care model is accepted by providers, patients, relatives and the population in the three rural target regions (Sulingen, Bad Gandersheim, Norden) and one urban area (Essen). The focus on rural areas is due to the fact that transformation processes of hospitals in rural areas are particularly sensitive politically, communally and publicly; the local hospital represents a central, sometimes identity-forming point of contact. It is therefore necessary to ascertain local circumstances and sentiments and to involve all stakeholders in the transformation process in a participatory manner. In addition, the findings on acceptance, needs, perspectives and challenges for the implementation of STATAMED are used to optimise the implementation process and create a basis for a successful transition to standard care.

2.5 Possible risks

Disadvantages, damage or violations of personal rights are not to be expected for the study participants.

Depending on the research method, the time required for the respondents is 1) 120 minutes for participation in a focus group 2) 30-60 minutes for participation in an interview 3) 180-240 minutes (but related to the observation in the facilities in total, does not count as interview time per respondent) 4) 120-150 minutes for participation in a discussion and information forum.

3. Characterisation of the study subjects

The aim of this study is to analyse the perspectives of healthcare providers, patients and their relatives as well as the population in the STATAMED target regions.

In principle, people of all genders (male/female) and ethnic backgrounds who are at least 18 years old can take part in the study.

Further specifics on inclusion and exclusion criteria, the intended sample sizes, the duration of the survey and the recruitment of respondents are differentiated depending on the sub-study.

(1) Focus group discussions with service providers involved and not involved in STATAMED Target group 1 are service providers involved in STATAMED, including: medical and nursing staff working in STATAMED facilities, patient guides/case managers, flying nurses and referring physicians in 4 STATAMED target regions (Essen, Norden, Bad Gandersheim, Sulingen).

In the 4 target regions to be evaluated, focus groups with service providers involved in STATAMED are to be conducted at 2 different points in time (up to one year after the start of the intervention; end of the intervention). Consequently, 2 focus groups with service providers are planned per location; 8-10 participants are to be interviewed per focus group. A total of 64-80 participants are envisaged (16-20 participants per location to be evaluated). Each focus group will last 120 minutes. Recruitment will take place in writing via an invitation letter; access and distribution of the letters will be facilitated by the respective pilot/case manager of the STATAMED centre.

Target group 2 are service providers not involved in STATAMED, including Clinicians in other hospitals, doctors in private practice, nursing staff from outpatient care services and inpatient care facilities who are not (yet) participating in STATAMED; who are located in the 4 target regions (Essen, Norden, Bad Gandersheim, Sulingen). In the 4 target regions to be evaluated, focus groups with service providers not involved in STATAMED are to be conducted at 2 different points in time (up to one year after the start of the intervention; end of the intervention). Consequently, 2 focus groups with service providers not previously involved in STATAMED are planned per location, with 8-10 participants to be interviewed per focus group. A total of 64-80 participants are envisaged (16-20 participants per location to be evaluated). Each focus group will last 120 minutes. Recruitment will take place in writing via invitation letters (e-mail, fax) or by telephone. The contact details of the regional providers will be researched manually, and the consortium management and partners will be contacted to add further facilities.

(2) Individual interviews with patients and relatives

The target group for the individual interviews are patients who were treated in STATAMED facilities (Essen, Norden, Bad Gandersheim, Sulingen) and their relatives if they were named by the patients as the main carer or person of trust and were involved in the care/support according to the person concerned. In addition to interest in the study, the prerequisites for participation in the interview are that the subjects are in good health and have sufficient knowledge of German to conduct an interview. Recruitment takes place in writing via an invitation letter; access and distribution of the letters is made possible via the respective pilot/case manager of the STATAMED centre.

(3) Participatory observation

In STATAMED, the processes and procedures are recorded on site as part of participant observation. The 4 STATAMED facilities to be evaluated in the regions of Esen, Norden, Bad Gandersheim and Sulingen are included. The on-site observation will take an estimated 3-4 hours.

to take advantage of this. The invitations are sent in writing and the appointments at the facilities are primarily arranged by telephone or email. The respective pilot/case manager of the STATAMED facility is responsible for accessing and distributing the letters.

(4) Discussion and information forums

The target groups for the discussion and information forums are the target groups from surveys 1-3 as well as the general population in the 4 target regions (Essen, Norden, Bad Gandersheim, Sulingen). One discussion and information forum with approx. 50-60 people is to be organised at each location. The event will last approx. 2-2.5 hours. Invitations will be sent out in writing via letters (e-mail, fax, post), information flyers, notices and social media. Several access strategies will be pursued for this wide-ranging target group: Distribution of invitations via contact persons at STATAMED institutions, manually researched distribution lists of providers, community foundations, consortium management networks and consortium partners. The distribution of flyers and posters is planned in various institutions, e.g. STATAMED facilities, medical supply stores, pharmacies, physiotherapy centres, city libraries.

4. Study type

STATAMED is a new cross-sector, general medical-short inpatient care model, which is being tested in a multi-centre setting at 6 locations and evaluated using a multi-method approach.

The qualitative process evaluation carried out by MHH is multi-perspective, prospective, retrospective and observational. Various qualitative methods (focus group discussions, individual interviews, participant observation, discussion and information forums) are used to determine the acceptance of different target groups, to identify factors influencing the implementation of the care model and to analyse changes in the day-to-day work of care providers as well as changes to care structures in the target regions.

5. Precise characterisation of the intended measures

The planning and implementation of the intervention is not part of the MHH's remit and is therefore only briefly outlined.

The new care model in STATAMED involves equipping a small clinic or separate ward with a general medical department (and, depending on regional requirements, a few surgical beds) without an emergency room. Each STATAMED facility has basic medical equipment, its own general practitioners and internists as well as nursing staff. Patients receive a structured referral consultation from the head physician, and treatment planning and interdisciplinary care is organised by a patient pilot as a case manager.

nager:in provides support. Specialised, mobile nursing staff (so-called "flying nurses") can be deployed in combination with telemedicine for follow-up care for up to 4 weeks after short-term inpatient treatment. The flying nurses carry out a medical assessment of the patient at home or in inpatient care facilities and can consult the STATAMED doctor via telemedicine. General practitioners and specialists can also provide post-inpatient care through consultation hours, home visits and non-medical practice assistance.

The resulting risks, burdens and safety precautions for patients and treatment providers must be assessed and accounted for by the consortium management (AOK Rheinland/Hamburg) and the consortium partners involved in the intervention (e.g. AOK Niedersachsen, SKH Stadtteilklinik Hamburg, Ubbo-Emmius Klinik Norden, Klinik Sulingen Landkreis Diepholz).

6. Measurements, findings and observations

The qualitative process evaluation consists of 4 sub-studies in which different qualitative research methods are used with different target groups.

6.1 Survey instruments

(1) Guided focus groups with service providers

At two different points in time (up to one year after the start of the intervention; end of the intervention), focus group discussions will be held in person or digitally with service providers who are involved in STATAMED (group 1) and service providers who are not involved in STATAMED (group 2). The focus group discussions are moderated with the help of guidelines. The guidelines will be developed by the MHH project team, and current findings from the surveys (questionnaire surveys, quality circles) of the consortium partners HCHE and HCB will also be taken into account during development. The core topics of the focus group discussions for Group 1 are Motivation to participate in STATAMED, facilitating and inhibiting factors in the implementation of STATAMED, changes in everyday working life, changes in care structures in the region. The guideline for group 2 contains the following areas: External perception of STATAMED, effects on care structures in the region, possible causes for non-participation in STATAMED (duration: 120 minutes).

(2) Guided individual interviews with patients and relatives

The individual interviews are conducted in person or by telephone using guidelines. The following main topics are addressed in the guidelines: Experiences in STATAMED, satisfaction with the care situation, involvement in decisions and communication

with carers. Patients and their relatives are also asked about their wishes, needs and opportunities for improvement (duration: 30 - 60 minutes).

(3) Participant observation in 4 STATAMED facilities

The participant observations are carried out in presence using a structured, criteria-led observation protocol. Relevant focal points are the processes involved in referral discussions and patient care, dealing with documentation systems, dealing with follow-up care and cooperation between providers in the interdisciplinary team and cooperation with referring physicians. On site, the project staff take care to influence events as little as possible (duration: 180 - 240 minutes).

(4) Discussion and information forums at 4 STATAMED locations

The discussion and information forums are based on guidelines. In addition to publicising the project and networking local stakeholders, the following topics are addressed in the discussion: (external) perception of STATAMED in the region; wishes, needs and opportunities for improvement, conditions for the continuation of STATAMED (duration 120 - 150 minutes).

6.2 Documentation of the measurements

The focus group discussions, individual interviews, discussion and information forums and conversations during the participant observation are recorded via audio device, transcribed (written down) and saved as a Word file or in rich text format (rtf). In addition, initial impressions and interim results of the focus group discussions and the information and discussion forums are recorded in writing and also visualised on metaplan cards or flipcharts.

The data from all transcripts and protocols are pseudonymised. Further details on this can be found in Section 9 on data collection and documentation.

The analysis is software-supported using MAXQDA, Word and Excel according to qualitative content analysis (Mayring or Kuckartz).

7. Detailed description of the study programme

The overall project is scheduled to run for 42 months and is divided into a preparatory phase (July 2023 - March 2024), intervention phase (April 2024 - March 2026), evaluation phase
(April 2026 - March 2027).

The planning, implementation and evaluation of the sub-studies of the qualitative process evaluation also extend over the entire project period. The most important work packages and milestones are summarised in the tabular overview (Fig. 1). In the preparatory phase of the qualitative process evaluation (July 2023 to March 2024), the documents for submission to the ethics committee will be prepared and the various guidelines for the qualitative sub-studies will be developed and piloted. The data collection and evaluation phase (April 2024 to December 2026) includes conducting the focus groups with service providers, individual interviews with patients and relatives, participant observation and discussion and information forums at the 4 sites to be evaluated, as well as qualitative content analysis of the data. Finally, the results of the qualitative process evaluation are combined with the results of other consortium partners in the project, i.e. with the quantitative data from the outcome and process evaluation (HCHE) and the scientific monitoring (HCB), in order to outline recommendations for the transition to rehabilitation care.

Aufgabe	Meilen stein	2023		2024				2025				2026				2027			
		Q3	*Q4*	*Q1*	*Q2*	*Q3*	*Q4*	*Q1*	*Q2*	*Q3*	*Q4*	*Q1*	*Q2*	*Q3*	*Q4*	*Q1*	*Q2*	*Q3*	*Q4*
Datenschutzkonzepte liegen vor	*6*		M6																
Positives Ethikvotum liegt vor	*7*		M7																
Entwicklung und Pilotierung der Befragungsinstrumente	*8*			M8															
Leitfaden für Fokusgruppen entwickelt und getestet																			
*Leitfaden für Interviews mit Patient*innen und Angehörigen entwickelt und getestet*																			
Leitfaden und Materialien für Diskussions- und Informationsforen vorbereitet																			
Beobachtungsprotokolle für teilnehmende Beobachtung vorbereitet																			
Vorbereitungsphase für die Evaluation und Begleitforschung abgeschlossen	*11*			M11															
Beginn Datenerhebung qualitative Prozessevaluation	*14*				M14														
Fokusgruppen 1 und 2 an allen vier Standorten durchgeführt																			
Diskussions- und Informationsforen an allen vier Standorten beworben und durchgeführt																			
Interviews mit Patient*innen und Angehörigen an allen Standorten geführt																			
Teilnehmende Beobachtungen an allen vier Standorten terminiert und durchgeführt																			
Fokusgruppen 3 und 4 an allen vier Standorten durchgeführt																			
Beginn Datenauswertung qualitative Prozessevaluation	*15*					M15													
Ende Datenerhebung qualitative Prozessevaluation	*24*											M24							
Ende Datenauswertung qualitative Prozessevaluation	*32*														M32				
Vorliegen Ergebnisse qualitative Prozessevaluation	*35*															M35			
Synthese der Ergebnisse und Empfehlungen für Übergang in Regelversorgung und alternativer Vergütungsansätze	*37*															M37			
Evaluationsbericht vorliegend	*38*															M38			

Figure 1 Work plan and schedule for the qualitative process evaluation

8. Target values

The qualitative process evaluation should provide insights into the following secondary endpoints:

- Acceptance of the new STATAMED care model by various target groups

- Degree of implementation: favourable and unfavourable factors influencing the implementation of STATAMED
- Changes in everyday working life at STATAMED facilities

Primary endpoints of the evaluation (e.g. re-hospitalisations and inpatient length of stay) are investigated by the consortium partner HCHE and are not part of this study.

9. Data acquisition and documentation

9.1 Data collection, input and access

The qualitative process evaluation data is recorded as described in section 6 on measurements, findings and observations.

The focus groups with service providers, individual interviews with patients and relatives, the participant observation in the facilities and the discussion and information forums at the 4 locations to be evaluated are conducted by the research assistant(s) from the project team, recorded with audio recording equipment and pseudonymised, i.e. transcribed in written form without naming names. The audio files are transcribed at the MHH either by project staff or by a professional transcription company. If transcription is carried out by a professional specialist company, a contract for commissioned data processing in accordance with Art. 28 GDPR is concluded in advance In addition, written protocols are prepared during the surveys and also pseudonymised. The transcripts and protocols are then imported into the qualitative data processing software MAXQDA (VERBI GmbH) and analysed by the research assistant.

9.2 Data security and data protection measures

Participation in the project is voluntary. All study participants agree to take part in the project via a written declaration of consent and receive study information with detailed information on data protection. All personal data in this study will be treated in accordance with the applicable data protection guidelines.

Confidentiality in the context of the scientific evaluation of the project is ensured by assigning an identification number to all audio recordings. The identification number and name of the participants are combined in a password-protected list. The list is stored separately from the data collected so that there is no link between the participants' personal information and the identification numbers. Declarations of consent are also stored separately from the data collected in a locked filing cabinet.

The data is saved on the MHH server in the STATAMED project folder on the P drive and provided with limited access rights. Files containing personal data are stored in a separate subfolder and password-protected. Access to the digital folders is restricted to the employees involved in the study.

The data will only be scientifically analysed with regard to the objectives stated in the application to the sponsor. This also applies to supplementary data analyses for project-related qualification work. All qualitative data is analysed in pseudonymised form, i.e. without the names of persons, institutions and locations. When publishing the research results or presenting the project at conferences, no information is presented that could reveal the identities of the participants. Data folders without personal information identifying the participants are stored after the study has been completed. In accordance with the American Psychological Association (APA) Code of Ethics, Section 8.14 Sharing Research Data for Verification [24], the principal investigator will not withhold unidentifiable data from other professionals who may wish to verify the author's conclusions. If other professionals wish to use the data collected in the project to answer new research questions, they must first obtain permission from the research group and the authors.

All documents that allow personal data to be assigned to identification numbers are deleted after ten years.

10. Adverse events

Disadvantages, damage or violations of personal rights are not to be expected for the study participants.

Depending on the research method, the time required for the test subjects is 1) 120 minutes for participation in a focus group 2) 30-60 minutes for participation in an interview 3) 180-240 minutes (counts as the total observation time in the facilities) 4) 120-150 minutes for participation in a discussion and information forum

The Institute of General Practice and Palliative Medicine at the MHH has also established an error conference. Should difficulties arise within the project team, these are addressed and discussed as part of the established error culture.

11. Evaluation/biometrics

The evaluation is carried out by the scientific staff of the Institute of General Medicine and Palliative Medicine.

The conversations during the focus group discussions, individual interviews, discussion and information forums and participant observation are recorded and transcribed using audio equipment,

pseudonymised and qualitatively analysed by several (at least two) researchers according to Mayring or Kuckartz using the MAXQDA program (VERBI GmbH). In addition, the protocols prepared are also analysed qualitatively.

12. Changes to the protocol

The final design of the guidelines for the 4 qualitative sub-studies and the design of the cover letters for the study subjects will be finalised in the further course of the project and submitted to the ethics committee. If the project team intends to amend the study protocol at a later date, this will be submitted to the ethics committee in advance.

13. Publication clause

In order to promote the accessibility and long-term preservation of research data and results, applicants will report comprehensively and transparently on the project and write national and international publications, if possible with open access, regardless of the results achieved. In addition, results will be presented at (inter)national conferences. In addition, applicants are prepared to provide digitised research data that is protected by data protection and copyright laws for secondary use in the event of justified requests.

The project team also has a great interest in making a visible contribution to the transfer of the results into care practice. The results of the project can provide new insights into how the new care model is accepted by different target groups, particularly in rural areas, and which factors influence the implementation process.

The G-BA Innovation Fund (project sponsor) has no influence on the design and execution of this study, on the collection, management, analysis of the data or interpretation of the results, or on the decision to publish the results.

14. References

Bertelsmann Foundation (2019) Overuse - a search for clues. DOI: 10.11586/2019064

Groening, M., Grossmann, F., Hilmer, T., Singler, K., Somasundaram, R., Wilke, P. (2017). Elderly emergency patients Deutsches Ärzteblatt 114(11): A-512 / B-446 / C-436.

Nowossadeck, E. (2012). Population aging and hospitalisation for chronic disease in Germany. Deutsches Ärzteblatt International, 109(9), 151.

Federal Statistical Office (2023a). *Demographic change: Proportion of the population aged 65 and over increased from 10% to 22% between 1950 and 2021.* Press release no. N033, available at https://www.destatis.de/DE/Presse/Pressemitteilungen/2023/06/PD22_N033_12.html [accessed on 02.11.2023].

Federal Statistical Office (2023b). *Care projection: 1.8 million more people in need of care expected by 2055.* Press release no. 124 of 30 March 2023, available at https://www.destatis.de/DE/Presse/Pressemitteilungen/2023/03/PD23_124_12.html [retrieved on 02.011.2023].
